# Exploring the influence of psychological factors on the comorbidity of dental caries and obesity in adolescents from the perspective of the oral-gut-brain axis

**DOI:** 10.3389/fcimb.2025.1659042

**Published:** 2025-09-16

**Authors:** Meng Wang, Xiaopeng Yang, Yimin Li, Mingxin Jiang, Bairu Chen, Wei Yang, Nan Ma, Shili He, Chengyue Wang

**Affiliations:** ^1^ Department of Prosthetics, Affiliated Stomatology Hospital of Jinzhou Medical University, Jinzhou, China; ^2^ Collaborative Innovation Center for Health Promotion of Children and Adolescents of Jinzhou Medical University, Jinzhou, China; ^3^ Department of Pedodontics, Affiliated Stomatology Hospital of Jinzhou Medical University, Jinzhou, China; ^4^ School and Hospital of Stomatology, China Medical University, Shenyang, China

**Keywords:** oral-gut-brain axis, caries, obesity, adolescents, microbiota

## Abstract

**Introduction:**

This study investigates how psychological factors influence the comorbidity of dental caries and obesity in adolescents through the oral-gut-brain axis. Adolescence is a critical period for both physical and psychological development, yet dental caries and obesity are prevalent issues that can negatively impact mental health. The study aims to provide insights into the underlying mechanisms and potential prevention and treatment strategies.

**Methods:**

An epidemiological survey was conducted on 1,024 students aged 12–15 from Beizhen No. 1 Junior High School. A total of 90 adolescents were selected for biosample research. The methods used included 16S rRNA gene sequencing, untargeted metabolomics, and SourceTracker analysis to examine oral and gut microbiota and metabolite concentrations.

**Results:**

Significant differences in oral and gut microbiota and metabolite concentrations were found among adolescents with different health statuses. Adolescents with caries and obesity showed distinct microbial profiles compared to healthy controls. The study also identified potential oral and gut microbial biomarkers associated with psychological disorders. SourceTracker analysis revealed a higher rate of ectopic colonization of oral microbiota in the intestines of adolescents with caries and obesity.

**Discussion:**

The study highlights the roles of the oral-gut and oral-brain axes in the comorbidity of dental caries and obesity among adolescents. The findings suggest that oral and gut microbiota play crucial roles in disease progression, and their imbalances may affect mental health through the oral-gut-brain axis. The results provide a theoretical foundation for developing integrated intervention strategies targeting both oral and systemic health.

## 1 Introduction

Adolescence represents a critical developmental window for both physical and psychological maturation; yet the concurrent “dual epidemic” of dental caries and obesity now threatens the health of this generation. The World Health Organization (WHO) 2022 report documents a 50–70% prevalence of permanent-tooth caries among 12- to 15-year-olds (Lobstein et al., 2015). According to the World Obesity Atlas 2023 and the 2025 Global Burden of Disease study in The Lancet, obesity prevalence among boys is projected to rise from 10% in 2020 to 20% by 2035, and among girls from 8% to 18% over the same period. The mental health landscape is equally alarming: UNICEF/WHO. State of the World’s Children 2021: On My Mind—Promoting, Protecting and Caring for Children’s Mental Health indicates that approximately 13–14% of children and adolescents worldwide suffer from psychological disorders, with prevalence rates for anxiety and depression increasing from 3.6% and 1.1% in 10- to 14-year-olds to 4.6% and 2.8% in 15- to 19-year-olds, respectively.

Dental caries, obesity, and psychological disorders do not occur in isolation; rather, they constitute a “networked disease complex” that reciprocally amplifies risk. Poor oral health undermines self-esteem and self-confidence, thereby precipitating anxiety and depression (Kisely et al., 2015). Conversely, individuals with compromised mental health—including severe mental illness, mood disorders, and eating disorders—often neglect oral hygiene (Kisely et al., 2016), which elevates the incidence of dental caries (Folayan et al., 2018). Emotional eating and stress-induced bingeing increase the intake of energy-dense foods, promoting obesity (Folayan et al., 2018). Obesity, in turn, reduces salivary flow and lowers oral pH, thereby enhancing caries susceptibility (Milaneschi et al., 2019). Carious lesions impair masticatory efficiency and nutrient absorption, further disrupting weight control (Chala et al., 2017).

Over the past five years, three converging “sub-axes” have provided the most recent empirical foundation for an integrated “oral–gut–brain axis.”Oral–gut axis: A systematic review by Yamazaki et al. demonstrated that periodontal pathogens—most notably Porphyromonas gingivalis—can be continuously swallowed with saliva, breach the intestinal barrier, and trigger local dysbiosis and systemic inflammation, thereby establishing the first direct mechanistic link between periodontitis and gut microbial imbalance (Yamazaki, 2023)Oral–brain axis: Bowland and Weyrich introduced the conceptual framework of the “oral–microbiome–brain axis,” emphasizing its relevance to neuropsychiatric disorders (Bowland and Weyrich, 2022). Dominy et al. identified P. gingivalis and its gingipains in post-mortem Alzheimer’s disease (AD) brain tissue, providing direct pathogenic evidence linking oral infection to neurodegeneration (Dominy et al., 2019).Gut–brain axis: Duan and Wu summarized how gut microbiota modulate serotonin (5-HT), brain-derived neurotrophic factor (BDNF), and microglial activity via short-chain fatty acids and tryptophan metabolites, thereby influencing the pathogenesis of depression and Parkinson’s disease (Duan and Wu, 2024). Furthermore, Zhang et al. reported that small-molecule inhibitors targeting oral pathogens can attenuate cerebral β-amyloid deposition, underscoring the therapeutic potential of cross-axis interventions (Zhang et al., 2023).

**Figure 1:**
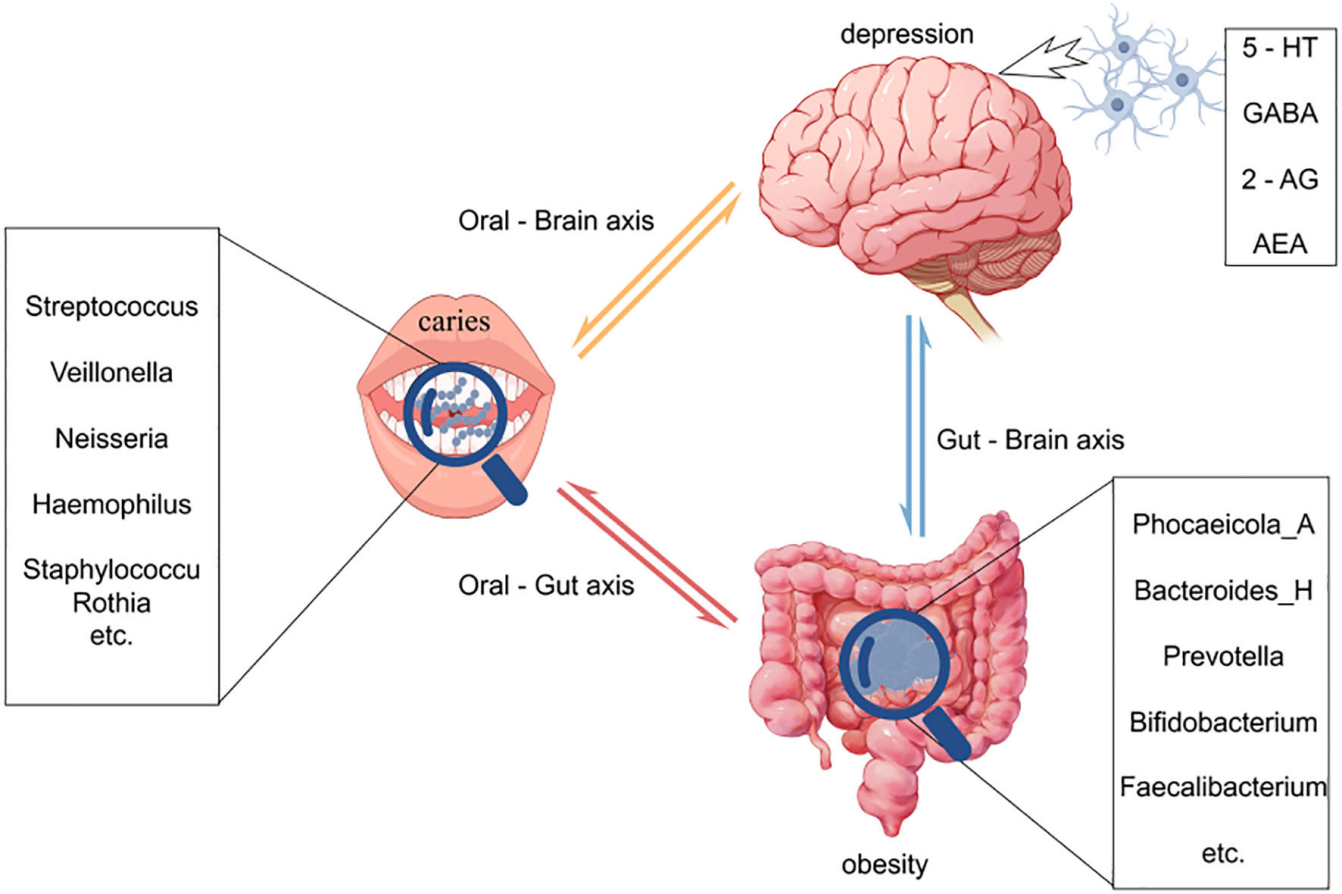
This figure illustrates the intricate interactions among the oral, gut, and brain axes and their effects on health. Oral microbiota directly associate with brain function through the oral-brain axis, potentially influencing the onset of neuropsychiatric disorders such as depression. Concurrently, oral microbes impact the gut microbiome via the oral-gut axis, subsequently modulating brain function and behavior through the gut-brain axis. Imbalances in oral microbiota (e.g., caries) and gut microbiota (e.g., changes associated with obesity) may affect the levels of neurotransmitters through these axes, thereby playing a role in the development of diseases like depression. This model underscores the dynamic connections among the oral, gut, and brain, highlighting their significance in overall health.

In summary, dental caries, obesity, and psychological disorders manifest as a syndemic among adolescents, and the emerging concept of the oral–gut–brain axis offers a unifying theoretical framework for elucidating their shared mechanistic underpinnings and for developing integrated intervention strategies (**Figure 1**).

## 2 Materials and methods

### 2.1 Survey subjects

In September 2024, an epidemiological survey was conducted on 1024 students from the First Junior High School in Beizhen City, Jinzhou City. After questionnaire surveys, physical parameter measurements, and oral examinations, a total of 90 adolescents aged 12–15 were included in the biological sample study.

#### 2.1.1 Sampling strategy and sample size calculation

This study employed a cross-sectional design with stratified cluster sampling. Fieldwork was conducted in the Beizhen district of Jinzhou City in September 2024. After selecting one fixed-point junior high school, we first stratified students by grade and then randomly selected intact classes within each stratum as primary sampling units.

Sample size was calculated according to the standard formula for cross-sectional surveys, *N=K × Q/P*, where K=400 when the permissible error is set at 10%. Using the most recent National Oral Health Epidemiological Survey (NOHES), the caries prevalence (P) for 12- and 15-year-olds was reported to be 38.5% and 44.4%, respectively; the larger value (44.4%) was adopted to yield a conservative estimate. This produced a minimum required sample of approximately 501 participants per group. To account for the design effect inherent in cluster sampling and an anticipated 10% non-response rate, the final sample was expanded to 1,024 adolescents, ensuring adequate statistical power and representativeness of the findings.

#### 2.1.2 Inclusion criteria for biological sample subjects

All subjects had not taken antibiotics within 1 month before the examination, had no systemic diseases, no congenital diseases or other diseases (oral mucosal diseases), and did not wear orthodontic appliances in the mouth. This study was approved by the Ethics Committee (Approval Number: JYFELL202409). The children examined obtained the permission of their parents or guardians and signed the informed consent form. A total of 90 participants were enrolled for biological sample collection in this study. The inclusion method is presented in the following **Table 1**:

**Table 1:** Biological sample collection criteria.

Stage	Procedure	Excluded (n)	Remaining (n)	Notes
Total Population	Students aged 12-15 from Beizhen No. 1 Middle School		1,024	Initial survey population
Stage 1: Screening	Excluded: antibiotic/probiotic use (within 1 month), systemic diseases, orthodontic appliances, etc.	187	837	Key exclusion criteria detailed in Section 2.1.2
Stage 2: Stratification	Grouped by health status			(criteria in Section 2.4):
–	• HG	–	180	No caries, obesity, or psychological disorders
–	• COG	–	125	Diagnosed with both caries and obesity
–	• PDG	–	17	No caries/obesity; diagnosed with psychological disorders
–	• COPG	–	44	Diagnosed with caries, obesity, and psychological disorders
Stage 3: Sampling	Each group was randomly selected			–
–	• The initial plan was 23 cases per group			Adjusted due to insufficient PDG sample size and depth issues
–	• Supplement to 30 cases per group			Random number table method
–	• Remove low quality samples (e.g. DNA extraction failure)			Final: 30 per group via random number table

### 2.2 Research methods

#### 2.2.1 Questionnaire survey

The demographic characteristics of the survey subjects, including the educational level of the parents of the survey subjects, the frequency of tooth brushing, the frequency of sweets and beverages, were collected through one-on-one questionnaire surveys. The International General Health Questionnaire-12 (GHQ-12) was used to assess the mental health status of students.

#### 2.2.2 Physical parameter measurement

Trained medical examiners measured the height, weight, and waist circumference. The height was measured using a metal column height meter, the weight was measured using an electronic weighing scale, and the waist circumference was measured using a non-elastic soft ruler with a minimum scale of 1 mm. The weight was accurate to 0.01 kg, and the height and waist circumference were accurate to 0.1 cm. They were recorded separately, and the body mass index of each survey subject was calculated.

#### 2.2.3 Dental caries examination

Using the methods and standards published by the World Health Organization, professional dentists used disposable oral instruments under a unified artificial light source, used probes and flat mirrors, and used a combination of visual and probing methods to examine and evaluate the dental health status of the survey subjects. A special recorder checked and recorded the caries, loss, and filling of each tooth.

### 2.3 Diagnostic criteria

#### 2.3.1 Psychological disorder

The GHQ-12 questionnaire consisted of 12 questions, using the Likert 4-point scoring method, with options ranging from 1 point “never” to 4 points “often”. The score ranged from 12 to 48, and the higher the score, the lower the mental health level. A total score > 27 indicated poor mental health.

#### 2.3.2 Dental caries

(1) Caries-free: No signs of caries and no fillings due to caries.

(2) Dental Caries: The pits or smooth surfaces of the teeth have softening at the bottom, potential damage to the enamel, or softening of the walls, which are divided into pit and fissure caries and smooth surface caries.

(3) Filled with Caries: Teeth with permanent fillings and one or more new caries.

(4) Filled without Caries: Teeth with one or more permanent fillings and no other caries.

(5) Missing due to Caries: Teeth lost or extracted due to caries.

#### 2.3.3 Obesity

(1) BMI(Body Mass Index): According to the Comprehensive Evaluation of the Development Level of Children and Adolescents (GB/T 31178-2014) standard (Comprehensive evaluation of children and adolescents development, n.d.), children and adolescents are divided into thin, normal, overweight, and obese. When the BMI of the investigator is less than the “normal” threshold point of the corresponding gender and age group, it is considered thin; when the BMI is less than the “overweight” threshold point of the corresponding gender and age group, it is considered normal; when the BMI is greater than or equal to the “overweight” threshold point of the corresponding gender and age group, it is considered overweight or obese. (BMI=Weight (kg)/[Height (m)]^2).

(2) Waist Circumference: According to the Screening Threshold for High Waist Circumference in Children and Adolescents aged 7–18 Years (WS/T 611-2018) (20180704145130574.pdf), the P90 of waist circumference in different gender and age groups is used as the judgment standard for central obesity.

### 2.4 Biological sample collection

All samples were divided into four groups, with 10 samples taken from each group

1. Healthy Group (HG)
2. Caries and Obesity Group (COG)
3. Psychological Disorders Group (PDG)
4. Caries, Obesity and Psychological Disorders Group (COPG)

### 2.5 Statistical methods

IBM SPSS Statistics 26.0 statistical software was used for data processing and analysis. Quantitative data that conformed to a normal distribution were described using (x¯ ± s), and the t-test was used for comparison between two groups, and analysis of variance was used for comparison between multiple groups. Qualitative data were described using the number of cases (%). The significance level α was set to 0.05 unless otherwise specified.

A one-way analysis of variance (ANOVA) was performed for each demographic variable (age, sex, BMI, etc.). **Table 2** presents the ANOVA results with the total psychological score as the dependent variable and caries status, BMI, age, sex, and comorbidity as independent variables. Participants with caries (n=205) exhibited significantly higher psychological scores than their caries-free counterparts (n=819, p < 0.001). A statistically significant age difference was also observed between the two groups (p=0.009). In contrast, neither BMI (22.15 ± 5.19 vs. 21.68 ± 5.14) nor sex distribution (1.51 ± 0.50 vs. 1.53 ± 0.50) differed significantly (p > 0.05). Notably, the caries–obesity comorbidity showed the most pronounced between-group difference (p < 0.001), indicating that the co-occurrence of caries and obesity may exert an additive effect on psychological distress.

**Table 2:** The aforementioned demographic variables—age, sex, BMI, and psychological status—will be included as covariates in subsequent microbiota analyses to control for their potential confounding effects on community structure.

Analysis of variance results				
Variable	Psychological total score evaluation	(Average ± standard deviation)		
	Psychology (*n*=205)	Normal (*n*=819)	*F*	*p*
Caries	0.74 ± 0.44	0.60 ± 0.49	13.773	0.000**
BMI	22.15 ± 5.19	21.68 ± 5.14	1.324	0.250
Age	13.49 ± 0.95	13.68 ± 0.97	6.815	0.009**
gender	1.51 ± 0.50	1.53 ± 0.50	0.267	0.605
Caries and obesity are common	1.52 ± 0.96	1.19 ± 1.02	17.665	0.000**

*p<0.05, **p<0.01.

## 3 Results

### 3.1 Basic characteristics

A total of 1,024 students were enrolled, comprising 483 (47.17%) males and 541 (52.83%) females. The majority were of Manchu ethnicity (n=813, 79.47%), followed by Han (n=197, 19.26%), Mongolian (n=8, 0.78%), and other ethnicities (n=5, 0.49%). Age distribution peaked at 13–14 years, accounting for 33.01% and 34.96%, respectively, with fewer participants in other age brackets. The overall caries detection rate was 62.50% (n=640). Regarding body-mass index (BMI), 499 participants (48.73%) were classified as having normal weight, 292 (28.52%) as obese, 83 (8.11%) as overweight, and 150 (14.65%) as underweight. Psychological assessment revealed that 819 individuals (79.98%) exhibited normal mental health status, whereas 205 (20.02%) presented with psychological or behavioral disorders (**Table 3**).

**Table 3:** The aforementioned demographic variables—age, sex, BMI, and psychological status—will be included as covariates in subsequent microbiota analyses to control for their potential confounding effects on community structure.

Essential information	Classify	Number (n)	Percentage (%)
	Han nationality	197	19.26
	Man nationality	813	79.47
Nation	Mongolian nationality	8	0.78
	Other nationality	5	0.49
Gender	Male	483	47.17
	Female	541	52.83
	11	1	0.10
	12	122	11.91
	13	338	33.01
Age	14	358	34.96
	15	188	18.36
	16	17	1.66
	No	384	37.50
Caries	Yes	640	62.50
	marasmus	150	14.65
	Normal	499	48.73
BMI	Overweight	83	8.11
	Obesity	292	28.52
evaluation	Psychology	205	20.02
Psychological	Normal	819	79.98
Total		1024	100.0

### 3.2 Demographic characteristics of participants providing biological samples

Of the 1,024 adolescents aged 12–15 years initially surveyed, four groups were established for biological sampling: the Healthy Group (HG, n=180), the Caries-Obesity Group (COG, n=125), the Psychological Disorders Group (PDG, n=17), and the Comorbid Caries-Obesity-Psychological Disorders Group (COPG, n=44). Mean age ranged from 13.30 to 14.59 years across the groups. BMI analyses revealed that the COG (29.11 kg/m²) and COPG (28.37 kg/m²) exhibited significantly higher values than the HG (19.45 kg/m²) and PDG (19.84 kg/m²), underscoring the co-occurrence of obesity and dental caries. Psychological assessment via the GHQ-12 (higher scores indicate poorer mental health) showed that the PDG (29.53) and COPG (28.95) displayed comparable and markedly elevated scores relative to the HG (17.73) and COG (18.60), thereby validating the diagnostic criteria used to define psychological morbidity in the grouping protocol (**Table 4**).

**Table 4:** Comparison of average age, BMI, psychological scores, and gender ratios across four groups: Healthy (HG), Caries-Obesity (COG), Psychological Disorders (PDG), and Comorbid Caries-Obesity-Psychological Disorders (COPG).

Group	Number (n)	average age	BMI average value	Psychological average score	Gender ratio is male to female
HG	180	14.33	19.45	17.73	81/99
COG	125	13.58	29.11	18.6	78/47
PDG	17	14.59	19.84	29.53	9/8
COPG	44	13.30	28.37	28.95	25/19

### 3.3 Sequencing information

All microbiota analyses were adjusted for age, sex, BMI, and psychological score to ensure that observed microbial differences are attributable to disease status rather than demographic characteristics.

**Figure 2:**
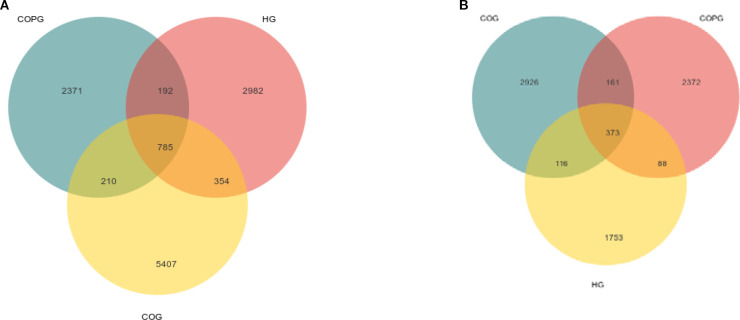
**(A, B)** Venn diagram illustrating the overlap and unique classifications among COG, HG, and COPG.

90 samples were divided into three groups, all samples were sequenced by Illumina platform, the results are as follows:

Saliva: Among them, all samples in HG, COG and COPG group contained 68543 ASVs (Amplicon Sequence Variant): 354 ASVs in HG and COG group; 192 ASVs in HG and COPG group; 210 ASVs in COG and COPG group; ASVs 2982 unique to HG group; ASVs 5407 unique to COG group; and ASVs 2371 unique to COPG group (**Figure 2A**).
Feces: All samples in HG, COG and COPG group contained 49126 ASVs: 116 ASVs in HG and COG group; 88 ASVs in HG and COPG group; 161 ASVs in COG and COPG group; ASVs 1753 unique to HG group; ASVs 2926 unique to COG group; ASVs 2372 unique to COPG group (**Figure 2B**).

### 3.4 Relative abundance of species at the phylum level

#### 3.4.1 Saliva

**Figure 3:**
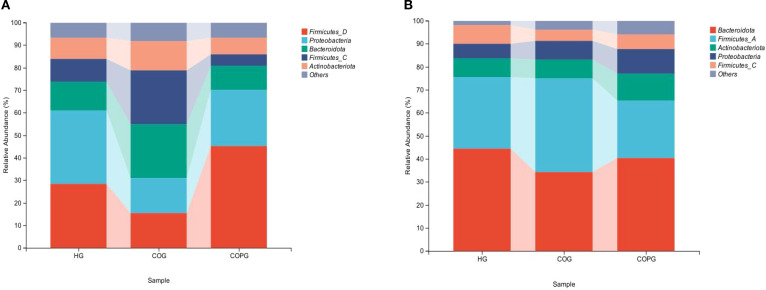
**(A, B)** Relative abundance of bacterial phyla across different samples at the phylum level.

The top five most abundant phyla in the saliva were selected, namely Firmicutes, Proteobacteria, Bacteroidota, and Actinobacteriota (**Figure 3A**). Compared with the HG group, the COG group exhibited higher relative abundance of Firmicutes, Bacteroidota, and Actinobacteriota, while the relative abundance of Proteobacteria was lower. In comparison with the COG group, the COPG group showed higher relative abundance of Firmicutes and Proteobacteria, and lower relative abundance of Bacteroidota and Actinobacteriota.

#### 3.4.2 Feces

The top five most abundant phyla in fecal samples were selected, namely Bacteroidota, Firmicutes, Actinobacteriota, and Proteobacteria, and a bar chart of their relative abundance was generated (**Figure 3B**). Compared with the HG group, the COG group exhibited higher relative abundance of Firmicutes and Proteobacteria, while the relative abundance of Bacteroidota and Actinobacteriota was lower. In comparison with the COG group, the COPG group showed higher relative abundance of Bacteroidota, Actinobacteriota, and Proteobacteria, and lower relative abundance of Firmicutes.

### 3.5 Relative abundance of species at the genus level

#### 3.5.1 Saliva

**Figure 4:**
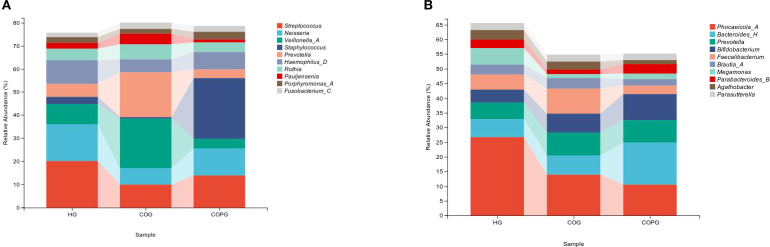
**(A, B)** Relative abundance of bacterial phyla across different samples at the genus level.

The most abundant genera were selected based on their relative abundance, and a bar chart of their abundance was generated (**Figure 4A**). Compared with the HG group, the COG group exhibited higher relative abundance of Veillonella, Prevotella, and Rothia, while the relative abundance of Streptococcus, Neisseria, Staphylococcus, and Haemophilus was lower. In comparison with the COG group, the COPG group showed higher relative abundance of Streptococcus, Neisseria, Staphylococcus, and Haemophilus, and lower relative abundance of Veillonella and Prevotella.

#### 3.5.2 Feces

The most abundant genera in fecal samples were selected based on their relative abundance, and a bar chart of their abundance was generated (**Figure 4B**). Compared with the HG group, the COG group exhibited higher relative abundance of Prevotella, Faecalibacterium, and Bifidobacterium, while the relative abundance of Phocaeicola and Megamonas was lower. In comparison with the COG group, the COPG group showed higher relative abundance of Bacteroides and Bifidobacterium, and lower relative abundance of Phocaeicola, Prevotella, and Faecalibacterium.

### 3.6 α-diversity analysis

α-diversity is an analysis of microbial diversity in community ecology, reflecting the distribution and differences of microbial communities (Miller et al., 2016).

**Figure 5:**
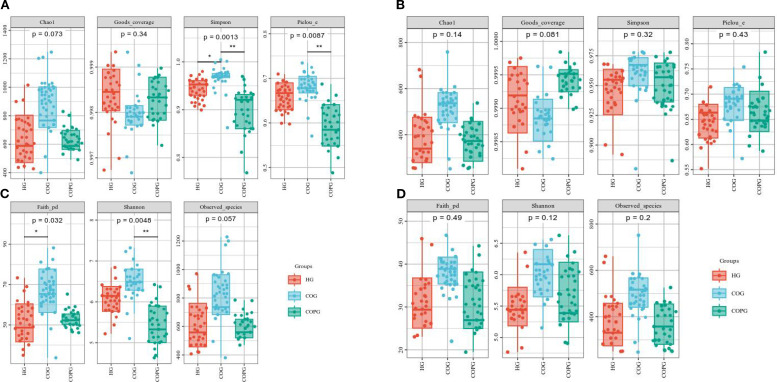
**(A–D)** Alpha-diversity analysis of oral and gut microbiota across different groups. All p-values were adjusted using the Benjamini–Hochberg FDR method; q < 0.05 was considered significant.

Oral microbiota α-diversity analysis (**Figure 5A**) revealed that the Simpson index in the COG group was significantly higher than that in the HG and COPG groups (P < 0.05). The Pielou_c and Shannon indices in the COPG group were significantly lower than those in the HG and COG groups (P < 0.05). Additionally, the Faith_pd index in the HG group was significantly lower than that in the COG and COPG groups (P < 0.05).

Gut microbiota α-diversity analysis (**Figure 5B**) showed that the Chao1, Simpson, and Observed species indices in the HG group were lower than those in the COG and COPG groups, although the differences were not statistically significant (P > 0.05). This indicates that there were no significant differences in the species diversity and richness of the gut microbiota among the groups.

### 3.7 β-diversity analysis

β-diversity is used to measure the similarity or difference among communities of different samples (Paudel et al., 2022).

**Figure 6:**
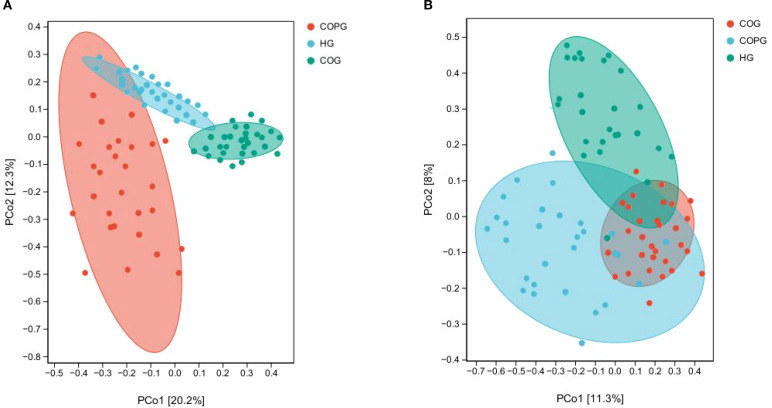
**(A, B)** Beta-diversity analysis of oral and gut microbiota across different groups.

The β-diversity differences among groups in saliva samples were assessed using Principal Coordinates Analysis (PCoA) methods (**Figures 6A, B**). To account for multiple comparisons, the p-values were adjusted using the Benjamini–Hochberg False Discovery Rate (FDR) method. The adjusted results indicated that the differences in microbial composition among the three groups (saliva and fecal samples) were significantly greater than the within-group differences, with all adjusted p-values being statistically significant (q < 0.05). This suggests that the oral and gut microbiota of healthy individuals, adolescents with dental caries and obesity, and those with psychological disorders exhibit distinct similarities and differences.

### 3.8 Analysis of microbial species differences

**Figure 7:**
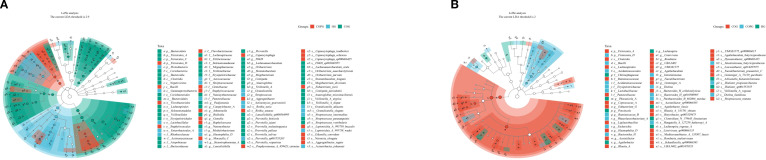
**(A, B)** LEfSe, analysis of microbial species differences among three groups.

LEFSe analysis (**Figures 7A, B**) was conducted to validate the differences in microbial species and identify potential oral and gut microbial biomarkers. To control for multiple comparisons, the p-values were adjusted using the Benjamini–Hochberg False Discovery Rate (FDR) method. Compared among the three groups, 114 species in the oral microbiota and 63 species in the gut microbiota exhibited significant differences (LDA > 2, q < 0.05).

In the oral microbiota, the HG group showed higher relative abundance of Neisseria and Porphyromonas. In contrast, the COG group exhibited significantly increased relative abundance of Fusobacterium, Rothia, and Prevotella. The COPG group had higher relative abundance of Streptococcus and Veillonella compared to the other groups.

In the gut microbiota, the HG group exhibited significantly higher relative abundance of Bacilli and Erysipelotrichaceae. The COG group had higher relative abundance of Firmicutes and Clostridia. These differentially abundant microbial taxa may play unique functional roles within their respective groups.

### 3.9 SourceTracker analysis

**Figure 8:**
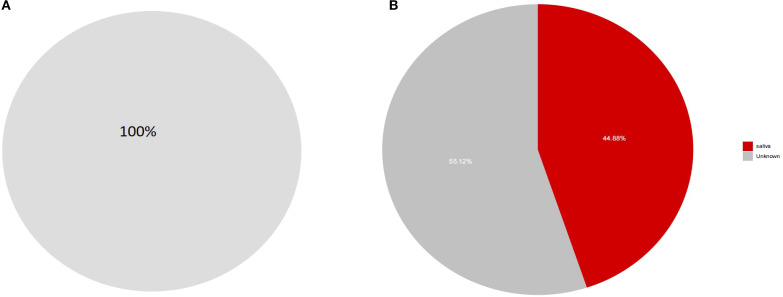
**(A, B)** SourceTracker analysis of ectopic colonization of oral microbiota in the intestines. These findings suggest that patients with dental caries and obesity have a higher rate of ectopic colonization of oral microbiota in their intestines compared to healthy individuals, which may have implications for understanding the microbial imbalances associated with these conditions.

To further investigate whether there is ectopic colonization of oral microbiota in the intestines of patients with dental caries and obesity, we employed the SourceTracker method for analysis(**Figure 8A, B**). The results demonstrated that nearly 44.88% of the Prevotella in the intestines of our experimental subjects originated from ectopic colonization of oral microbiota, which was higher than the nearly 100% result observed in the healthy population in this study.

### 3.10 Untargeted metabolomics analysis

**Figure 9:**
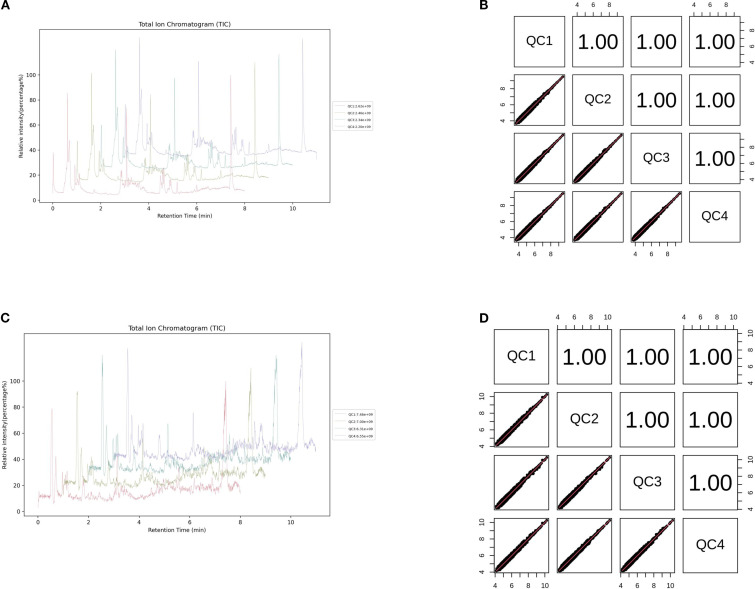
**(A–D)** Untargeted metabolomics analysis and quality control assessment.

This study conducted a comprehensive identification and quantitative analysis of metabolites in intestinal samples using untargeted metabolomics. Quality control (QC) analysis ensured the reliability and stability of the experimental data. The total ion chromatogram (TIC) of QC samples showed that the response intensity and retention time of each chromatographic peak were largely overlapping, indicating minimal variation caused by instrument error. The Pearson correlation coefficients between QC samples were all greater than 0.9, demonstrating excellent experimental reproducibility (**Figures 9A–D**).

**Figure 10:**
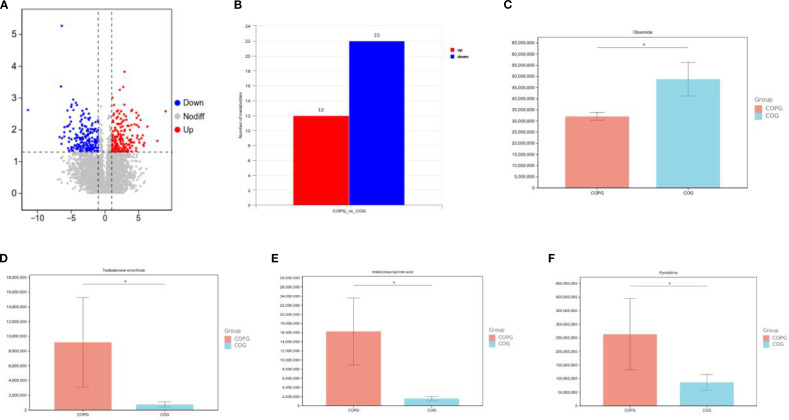
**(A–F)** Metabolite profile differences between COG and COPG groups in fecal samples.

In the fecal sample comparison between the COG and COPG groups, significant differences in metabolite profiles were observed, with 12 metabolites upregulated and 22 downregulated in the COPG group compared to the COG group. To account for multiple comparisons, the p-values were adjusted using the Benjamini–Hochberg False Discovery Rate (FDR) method. Oleamide was found to be significantly lower in the COPG group than in the COG group (q < 0.05). In contrast, testosterone enanthate, imidazolepropionic acid, and pyrrolidine were significantly elevated in the COPG group (q < 0.05) (**Figures 10A–F**).

**Figure 11:**
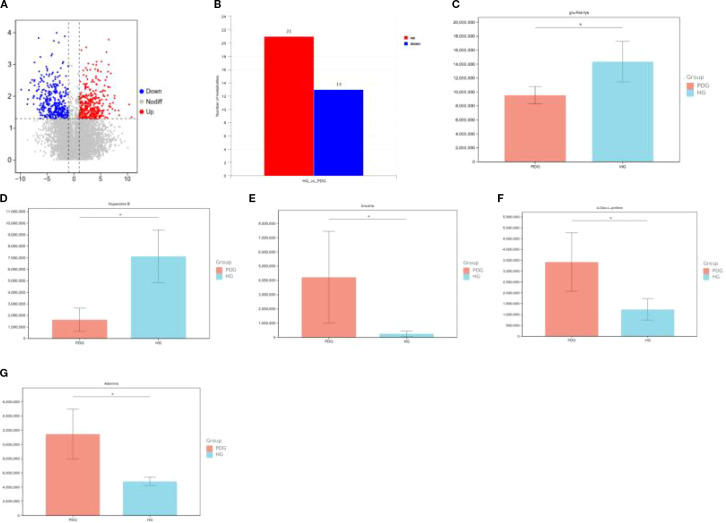
**(A–G)** Metabolite profile differences between HG and PDG groups in saliva samples.

In the comparison of saliva samples between the HG and PDG groups, significant differences in metabolite profiles were observed, with 21 metabolites upregulated and 13 downregulated in the PDG group compared to the HG group. To account for multiple comparisons, the p-values were adjusted using the Benjamini–Hochberg False Discovery Rate (FDR) method. Specifically, Glu-His-Lys (glutamine-histidine-lysine) and Huperzine B were found to be significantly lower in the PDG group than in the HG group (q < 0.05). In contrast, brucine, 4-oxo-L-proline, and adenine were significantly higher in the PDG group compared to the HG group (q < 0.05) (**Figures 11A–G**).

### 3.11 16S rRNA gene sequencing combined with non-targeted metagenomics analysis

In the integrated analysis of 16S rRNA gene sequencing and non-targeted metabolomics between the dental caries-obesity group and the dental caries-obesity-mental disorder group, a connection between microorganisms and metabolites associated with psychological issues was identified:

## 4 Discussion

### 4.1 Oral microbiome characteristics of the adolescent caries-obesity-psychological disorder triad

The oral cavity, as the initial part of the digestive tract communicating with the external environment, serves as the entry point for microorganisms into the human body, including bacteria, fungi, viruses, mycoplasmas, and other biological entities. The oral microbiota represents one of the most diverse and unique microbial communities in the human body, constituting the second most complex microbial ecosystem after the digestive system microbiota (Al Bataineh et al., 2022). Saliva samples, being readily obtainable, effectively represent microbial community information from various oral sites (Leake et al., 2016; Soriano-Lerma et al., 2020) and are widely utilized in oral microbial ecology research (Kaczor-Urbanowicz et al., 2017; Borkent et al., 2020). Next-generation sequencing (NGS) technology has emerged as a powerful tool for understanding the oral microbiome in health and disease states (Johansson et al., 2016; Al-Hebshi et al., 2019). By targeting one or more hypervariable regions of the 16S rRNA gene, this method characterizes microbial communities, as these variable regions serve as effective markers for identifying bacterial taxa in samples. In recent years, this approach has been extensively applied in studies investigating cariogenic microbial communities (Magana et al., 2018), enhancing our understanding of microbial factors associated with dental caries. Dental caries arises from the interplay among oral microorganisms, host factors, dietary components, and temporal influences (Pitts et al., 2021; Song et al., 2024), with oral microbiota playing a pivotal role in caries initiation and progression. While the potential connections between caries and systemic health remain underexplored (Chapple et al., 2017), current evidence suggests a stronger association between obesity and caries compared to other systemic conditions (Sabharwal et al., 2021). Our research group has previously demonstrated a positive correlation between caries prevalence and obesity. Obesity, a complex metabolic disease, has been linked to the oral microbiota. Recent studies have uncovered potential connections between them. The oral microbiota in obese individuals has been shown to change, which may promote systemic inflammation and metabolic disruption. The oral microbiota can influence the host’s metabolism through various mechanisms, such as producing short-chain fatty acids (SCFAs) and regulating immune responses. A 2025 study investigated the effect of orally administered probiotics on weight and found that certain probiotics could significantly reduce the weight and visceral fat levels of obese patients, indicating that the oral microbiota may play a role in weight management (Guo et al., 2025). Moreover, studies have shown that the composition of the oral microbiota can affect the gut microbiota, which in turn impacts metabolic health. For example, specific oral bacteria, such as Prevotella, have been associated with obesity-related changes in the gut microbiota. These findings highlight the importance of considering the oral microbiota in research on obesity and its related metabolic diseases. Concurrently, mental health issues exhibit high prevalence among adolescents (Gulliver et al., 2010; Guo et al., 2025), affecting approximately 10–20% of children and adolescents worldwide (Kieling et al., 2011; Aarons et al., 2019). The role of the oral microbiome in mental health has recently been appreciated within the proposed oral-brain axis. A 2024 study published in Nature found that periodontitis, a chronic bacterial infection, is associated with mental disorders such as depression and anxiety (Malan-Müller et al., 2024). This suggests the existence of an “oral-brain axis” and highlights the potential role of the oral microbiome in mental health.

In the oral microbiota of this study, adolescents with caries and obesity showed distinct microbial profiles compared to healthy controls at the phylum level: higher relative abundances of Firmicutes, Bacteroidota, and Actinobacteriota, and lower relative abundance of Proteobacteria, consistent with findings by Agnello et al (Advances in research on oral microecology and caries prevention, n.d.; Agnello et al., 2017; De Lemos et al., 2024). At the genus level, increased relative abundances of Veillonella, Prevotella, and Rothia were observed, while Streptococcus, Neisseria, Staphylococcus, and Haemophilus exhibited reduced abundances. Multiple studies (Mashima and Nakazawa, 2014; Do et al., 2015; Liu et al., 2020; Korona-Glowniak et al., 2021)have identified strong associations of Veillonella, Prevotella, and Streptococcus with dental caries. Notably, statistical analyses of Prevotella abundance in prior studies revealed its dominance in Western populations’ oral microbiota, with high-fiber diets and obesity linked to its enrichment (Tett et al., 2021). The reduced relative abundance of Streptococcus in the COG group may be attributed to the altered dietary habits and lifestyle in obese individuals, which may lead to changes in saliva composition, such as variations in nutrient content, and alterations in saliva flow rate. These changes may create an environment unfavorable for the growth and colonization of Streptococcus, thereby reducing its relative abundance. While some studies associate Neisseria with caries (Zheng et al., 2018), others report its prominence in caries-free groups (Richards et al., 2017). Baker et al (Baker et al., 2021), using next-generation whole-genome sequencing to compare salivary microbiota in American children (>3 years old) with and without caries, observed higher abundances of Neisseria and Haemophilus in caries-free groups, aligning with the findings of this study.

In adolescents with psychological disorders compared to psychologically normal individuals at the phylum level, Firmicutes and Proteobacteria exhibited higher relative abundances, while Bacteroidota and Actinobacteriota showed lower abundances. The reduced abundance of Actinobacteriota was associated with anxiety states and cortisol levels in adolescents (Simpson et al., 2020), potentially serving as a marker of hypothalamic-pituitary-adrenal (HPA) axis hyperactivity, a common feature in the pathophysiology of depression. At the genus level, Streptococcus, Neisseria, Staphylococcus, and Haemophilus displayed higher relative abundances, whereas Veillonella and Prevotella demonstrated lower abundances. The observed associations between these microbial taxa and psychological disorders align with findings from studies by Yolken R et al (Prato et al., 2021; Wingfield et al., 2021; Yolken et al., 2021; Al Bataineh et al., 2022).

The results of alpha diversity analysis align with findings from Liu et al (Liu et al., 2019), a phenomenon potentially associated with oral microbial dysbiosis. According to the “ecological plaque hypothesis,” oral microbiota maintains equilibrium under healthy conditions, but alterations in the oral environment disrupt this balance, driving the transition of symbiotic microbial communities into pathogenic biofilms, ultimately leading to dysbiosis and caries development (Advances in research on oral microecology and caries prevention, n.d.). Beta diversity analysis revealed significant differences in oral microbiota between healthy individuals and adolescents with caries-obesity or psychological disorders, underscoring the critical role of oral microbial communities in both oral and systemic health. Concurrently, LefSe results demonstrated distinct compositional differences in salivary microbial communities across groups, likely attributable to specific oral health statuses or lifestyle factors within each cohort. In the COPG group, enriched Streptococcus and Veillonella genera—early colonizers and typical symbionts in the oral cavity—jointly participate in the formation of early-stage biofilms. Extensive research indicates that dysbiosis of Streptococcus and Veillonella is not only closely linked to oral diseases such as caries and periodontitis but may also breach gastrointestinal barriers for distal colonization, emerging as novel potential biomarkers for predicting the onset, progression, and prognosis of various systemic diseases.

### 4.2 Characteristics of the gut microbiome in adolescents with the caries-obesity-psychological disorders triad

The gut microbiota is one of the most complex and diverse microbial communities in the human body, with its diversity even surpassing that of the oral microbiota, making it the most diverse among human microbial communities (Li et al., 2024). These microbes, including bacteria, fungi, viruses, and archaea, form a complex ecosystem that plays a crucial role in human digestion, immunity, and metabolism. Notably, B. acidifaciens, typically associated with the gut microbiota, has also been found to play a role in the oral microbiota of children with severe dental caries, indicating a possible link between gut and oral microbiota in the development of dental caries (Eriksen et al., 2024). Another study highlighted that children with poor oral hygiene and high caries rates showed an increased abundance of specific bacterial phyla and genera, which may disrupt the balance of the gut microbiota and affect systemic health (Ribeiro and Paster, 2023). Numerous studies comparing the gut microbiota of overweight and normal-weight children through 16S rRNA gene sequencing have found significant differences in microbial diversity and composition between the two groups (Pan and Jiao, 2024). Moreover, there is also a certain relationship between the gut microbiota and mood disorders, with research indicating that the relative abundance of certain bacteria changes in individuals with depression (Marano et al., 2025).

In the gut microbiota at the phylum level, adolescents with caries and obesity exhibited an elevated Firmicutes/Bacteroidota (F/B) ratio—characterized by increased Firmicutes and decreased Bacteroidota —a critical alteration implicated in the pathogenesis of obesity and related metabolic complications (Gallardo-Becerra et al., 2020). Concurrently, the reduced relative abundance of Actinobacteriota aligns with findings from Rocío et al (Quiroga et al., 2020), further supporting the strong association between gut microbial shifts and the caries-obesity phenotype. Notably, adolescents with caries, obesity, and psychological disorders demonstrated distinct microbial profiles compared to their psychologically normal counterparts with obesity: lower Firmicutes abundance and higher relative abundances of Bacteroidota, Actinobacteriota, and Proteobacteria, consistent with studies by Huang, Plaza-Díaz J, and colleagues (Huang et al., 2018; Jiang et al., 2018; Plaza-Díaz et al., 2019).

At the genus level, adolescents with caries and obesity showed higher relative abundances of Prevotella, Faecalibacterium, and Bifidobacterium, in agreement with findings by Moran-Ramos S et al (Moran-Ramos et al., 2023), alongside reduced abundances of Faecalibacterium and Megamonas. In contrast, those with psychological disorders displayed elevated Bacteroides and Bifidobacterium abundances but lower Faecalibacterium, Prevotella, and Faecalibacterium levels. These observations align with prior studies reporting reduced Prevotella abundance during depressive states (Simpson et al., 2021) and diminished Faecalibacterium levels in individuals with anxiety, depression, bipolar disorder, psychosis, and schizophrenia (Nikolova et al., 2021).

Alpha Diversity Analysis: The observed differences lacked statistical significance, which partially aligns with findings from (Pinart et al., 2021). This meta-analysis incorporated 32 cross-sectional studies evaluating gut microbial composition in obese and non-obese adults via high-throughput sequencing. Among these, 7 out of 22 studies reported no significant differences in alpha diversity. This phenomenon may be attributed to similarities in dietary patterns and habits among study participants, leading to consistent nutrient availability for gut microbiota. Such uniformity likely provides comparable ecological niches for microbial taxa, promoting stable microbial community diversity and minimizing alpha diversity variations.

### 4.3 Microbiota-gut-brain axis

**Figure 12:**
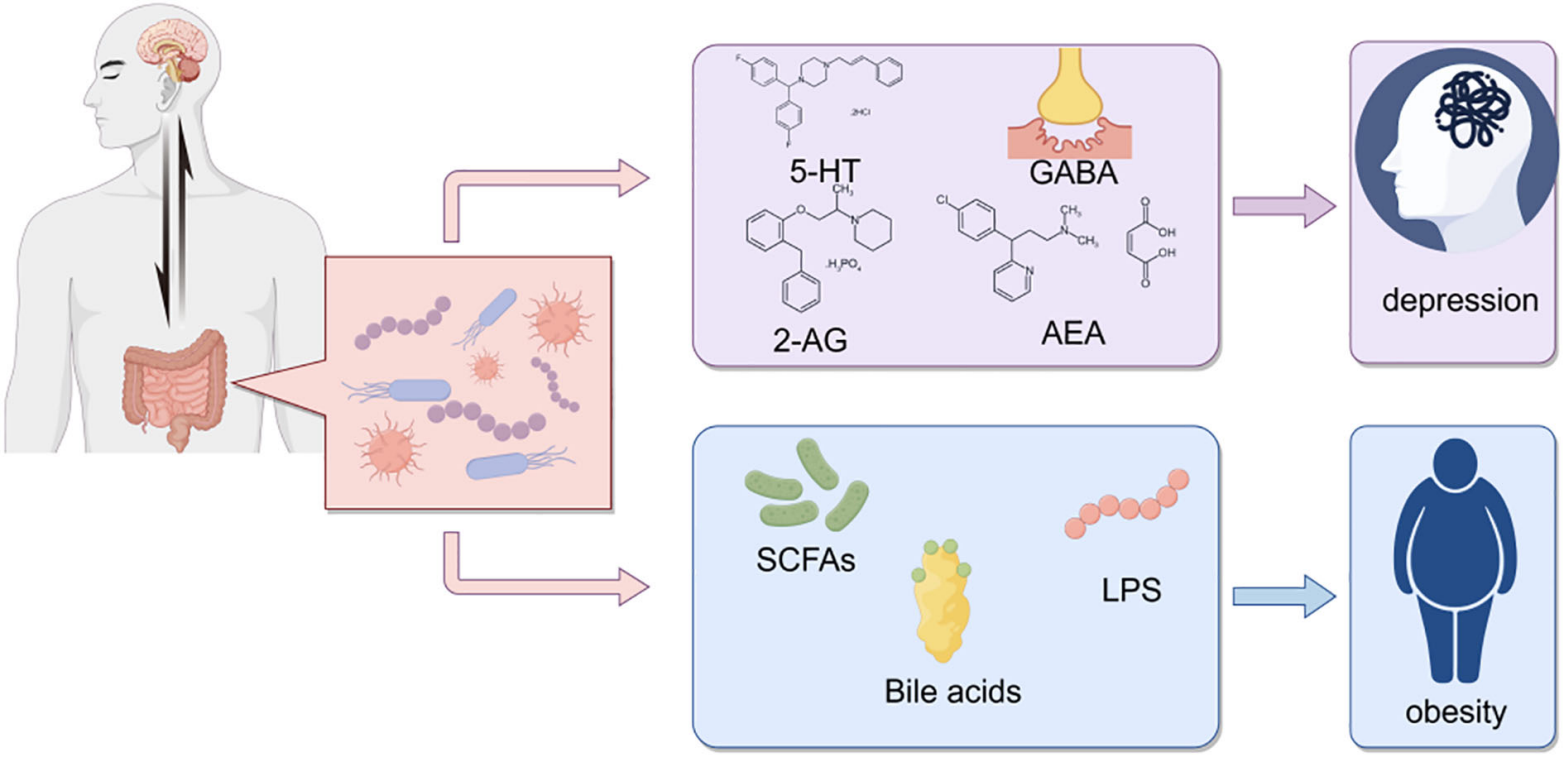
Interactions between gut microbiota and host health. This schematic diagram illustrates the complex interactions between gut microbiota and host health, highlighting the potential roles of various microbial metabolites in influencing neurological and metabolic conditions.

Communication between the gut and brain is bidirectional, involving multiple pathways such as neural, endocrine, and immune mechanisms (**Figure 12**). The microbiota and its derived metabolites act as critical regulators in gut-brain signaling, leading to the concept of the microbiota-gut-brain (MGB) axis (Cryan and Dinan, 2012). The gut-brain axis, a bidirectional communication system between the gut and the central nervous system mediated via neural, endocrine, and immune pathways, has emerged as a pivotal framework for studying interactions between gut microbes and neurological functions in recent years (Cryan and Dinan, 2012).

Analysis of fecal samples from the COG and COPG groups revealed significant alterations in multiple metabolite levels, which may exert broad effects on brain function via the gut-brain axis. Oleamide, which exhibits antidepressant-like properties in rat models, modulates neurotransmitter systems (e.g., 5-HT and GABA), and its decreased levels may impair mood regulation (Boger et al., 1998; Ge et al., 2015). Additionally, a Japanese study reported psychological changes, such as emotional instability, in some transgender males undergoing gender-affirming hormone therapy with testosterone enanthate, suggesting its potential influence on mood regulation via neuroendocrine pathways (Kirisawa et al., 2021). Imidazolepropionic acid, a histidine-derived microbial metabolite, may participate in neurological regulation by affecting neuroinflammation and neuronal function in the brain (Caradonna et al., 2024). Pyrrolidine, through its substituted derivatives, enhances 5-HT1A receptor potency by approximately 12-fold, indicating its potential role in modulating neurotransmitter system activity via 5-HT1A receptor regulation (Warren et al., 2024).

These findings further support the hypothesis that gut microbial metabolites may regulate brain function via the HPA axis (Foster and McVey Neufeld, 2013) and influence neuroinflammation, neurotransmitter systems, and mood regulation through the gut-brain axis, thereby exerting broad impacts on cerebral function. The results provide novel insights into the interactions between gut microbiota and the nervous system, suggesting that microbial metabolites could serve as potential therapeutic targets for future neuropsychiatric interventions.

Gln-Trp (Glutamine-Tryptophan): Tryptophan, a precursor to the neurotransmitter serotonin (5-hydroxytryptamine), is crucial for regulating emotions, sleep, and appetite. Glutamine, involved in various metabolic processes and important in the nervous system, associates with psychological factors through substance metabolism and neurotransmitter synthesis.

(2Z)-2-Benzylidenesuccinic Acid: As a phthalate compound, it is prevalent in daily life and may disrupt the endocrine system. Some studies indicate that exposure to phthalates is linked to neurological abnormalities, anxiety, and depression. A U.S. study measured phthalate metabolites in the urine of 153 pregnant women and found that higher mono-isobutyl phthalate levels in boys’ urine were associated with increased scores for inattention, rule-breaking, aggression, and conduct problems. Higher MBzP levels correlated with elevated scores for defiant behavior and conduct problems in boys.

Gln-Trp was positively correlated with Pseudoxanthomonas, suggesting Pseudoxanthomonas might influence psychological disorders. Conversely, Limivivens, Paraprevotella, Acetatifactor, and Eubacterium showed negative correlations, indicating their abundance might link to psychological issues. Notably, Paraprevotella, part of the Prevotellaceae family, warrants further attention.

### 4.4 Oral-brain axis

**Figure 13:**
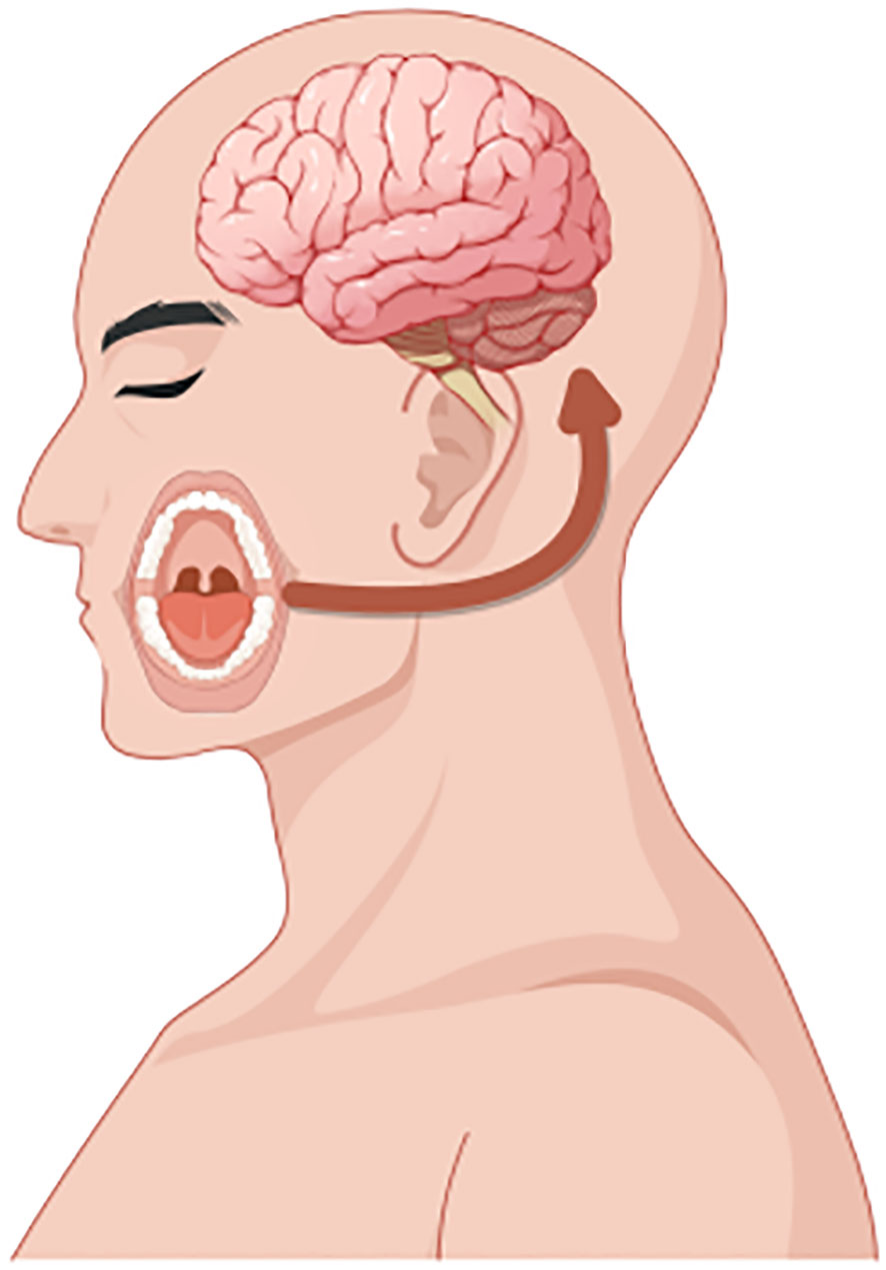
Schematic representation of the oral-brain axis. This schematic serves as a conceptual framework for understanding the complex interactions that may occur between oral microbiota, oral health conditions, and brain health, including the potential impact on cognitive functions and neurological disorders.

The mechanisms linking oral microbiota and the brain (termed the “oral-brain axis”) remain largely unexplored (Yano et al., 2015; Valles-Colomer et al., 2019). However, recent studies have proposed several potential routes through which oral bacteria may reach the brain and influence neuroimmune activity and inflammation (Olsen and Hicks, 2020). For instance, routine dental procedures such as flossing, brushing, and professional cleanings may introduce oral bacteria into the bloodstream, causing bacteremia (Olsen, 2008). Certain microbes might subsequently traverse the blood-brain barrier. Additionally, altered blood-brain barrier permeability could expose the brain to bacterial metabolites, triggering inflammatory responses that disrupt central nervous system function and contribute to psychological disorders such as anxiety and depression (**Figure 13**).

Analysis of differential metabolites in salivary samples from the HG and PDG groups revealed significant alterations in multiple metabolite levels. Notably, the glutamine-histidine-lysine (Glu-His-Lys) tripeptide, composed of three amino acids, exhibited notable changes. Glutamate (Glu), a critical excitatory neurotransmitter in the brain, is essential for normal cognitive functions such as learning, memory, and attention. However, imbalanced glutamate levels are strongly associated with the pathogenesis of psychiatric disorders (The structure, function and role in neuropsychiatric disorders of glutamate transporters, n.d.), where excessive concentrations may contribute to depression, anxiety, and schizophrenia. Magnetic resonance spectroscopy (MRS) studies further highlight glutamate’s potential role in conditions like depression and schizophrenia through quantitative measurements of its cerebral concentrations (The role of the glutamate system in mental disorders: Based on magnetic resonance spectroscopy imaging technology, n.d.). Histidine (His), a precursor of histamine, serves as both a neurotransmitter and neuromodulator in the brain, regulating sleep-wake cycles, feeding behavior, and learning (Ma et al., 2023). Clinical and experimental studies have elucidated histamine’s role in neuropsychiatric disorders (Cheng et al., 2021). Specifically, histamine binds to inhibitory receptors on murine serotonergic neurons, directly suppressing serotonin (5-hydroxytryptamine, 5-HT) release—a key molecule in mood regulation (Hersey et al., 2021). Reduced serotonin levels are closely linked to adverse psychological states such as depression and anxiety. Lysine (Lys), an essential amino acid involved in protein synthesis, also contributes to serotonin production (Yang et al., 2018). By enhancing serotonin availability, lysine may modulate mood regulation and potentially alleviate anxiety symptoms. Additionally, Huperzine B—a natural alkaloid derived from Huperzia species—exhibits potent acetylcholinesterase (AChE) inhibitory activity, demonstrating therapeutic potential for Alzheimer’s disease (Knölker, 2013). This suggests its applicability in managing psychological disorders associated with cholinergic dysfunction, such as Alzheimer’s disease.

Brucine intoxication may increase neuroexcitability, leading to muscle spasms and seizures, symptoms potentially linked to psychological states such as anxiety and tension (Lu et al., 2020). Direct associations between 4-Oxo-L-proline and psychological disorders remain understudied; however, its parent compound, L-proline, has been extensively investigated for its role in the nervous system and the relationship between its metabolic abnormalities and psychiatric conditions. Elevated concentrations of L-proline derivatives may exert toxic effects on the nervous system, such as inducing seizures or impairing normal neuronal function (Das et al., 2025). Studies have highlighted the role of proline metabolism in neuronal activity, suggesting that its dysregulation may contribute to neurological dysfunction (Yao and Han, 2022), thereby increasing susceptibility to neuropsychiatric disorders, including depression and anxiety. Adenine, a key component of purine metabolism, produces adenosine as a metabolite that plays a vital regulatory role in the nervous system. Research indicates that adenosine and its receptors (particularly the A2A subtype) are critically involved in mood regulation and mood disorders. In individuals with depression, adenosine metabolism may be disrupted, and the activity of its receptors (e.g., A2A) is closely associated with emotional behaviors (Qi et al., 2025).

In summary, the interaction between oral microbiota and the brain (termed the “oral-brain axis”) may play a significant role in mental health. Oral bacteria and their metabolites can influence the central nervous system through multiple pathways, including direct penetration of the blood-brain barrier, activation of immune responses, or signal transmission via neural routes. Dysbiosis of the oral microbiota is closely associated with psychiatric disorders such as anxiety and depression, highlighting oral health as a critical determinant of mental well-being. These insights not only deepen our understanding of the oral-brain axis but may also inform novel strategies for clinical interventions targeting psychiatric conditions.

### 4.5 Oral-gut axis

**Figure 14:**
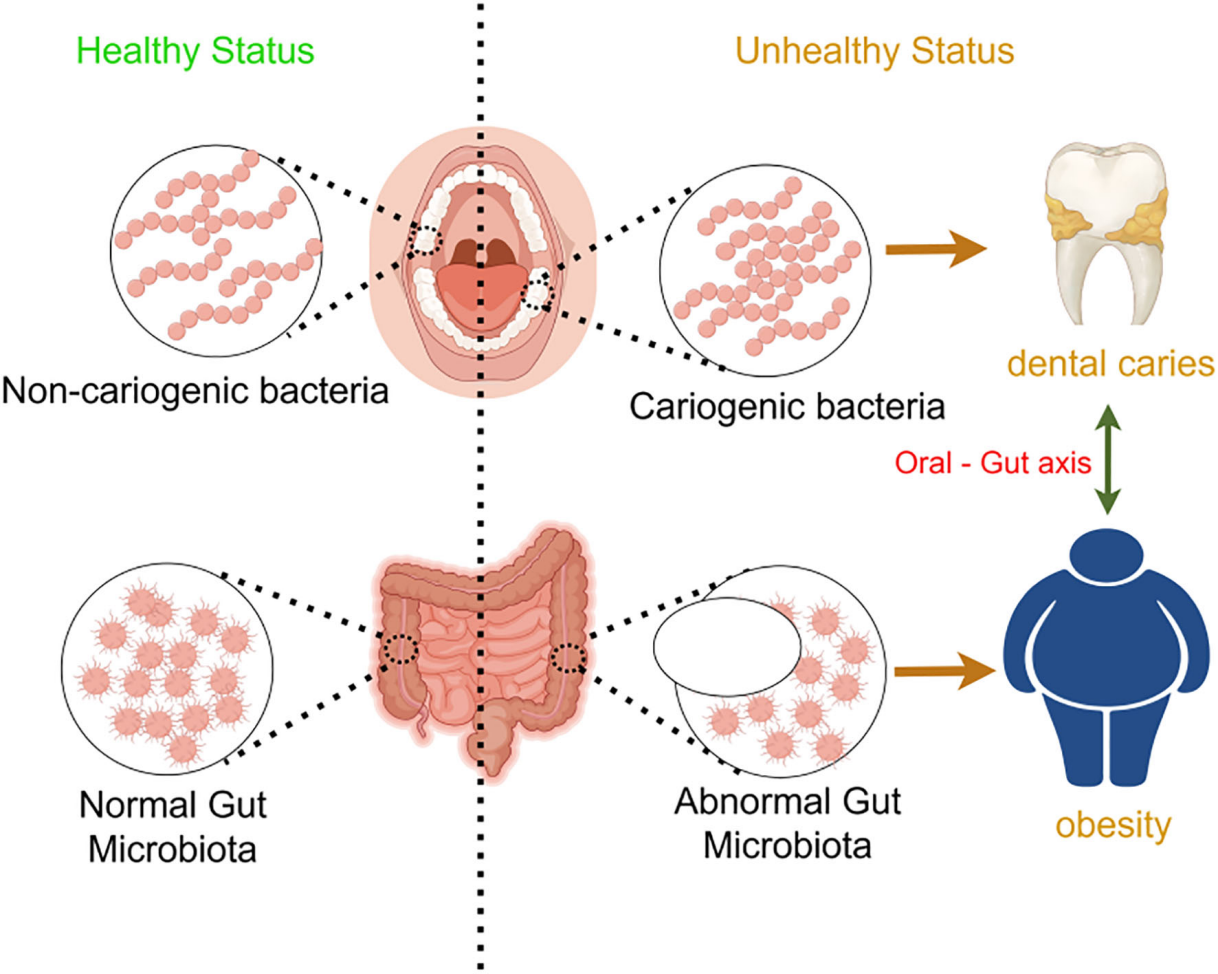
Schematic representation of the oral-gut axis and its impact on health. This schematic highlights the importance of maintaining a healthy oral and gut microbiota to prevent dental diseases and metabolic disorders, emphasizing the role of the oral-gut axis in overall health.

The oral-gut barrier, primarily shaped by the physical distance between the oral cavity and gut as well as differences in their chemical environments, allows each niche to harbor distinct microbial communities (Paudel et al., 2022). A substantial number of oral bacteria are swallowed in saliva, but the low pH of gastric acid creates harsh chemical conditions that prevent many oral bacteria from colonizing the gut (**Figure 14**). However, various factors, including disease, medication, and aging, may facilitate ectopic colonization of oral bacteria in the gut (Park et al., 2021). Oral bacteria can translocate to the gut and alter gut microbiota composition. The linkage between oral pathogenic bacteria and gut microbiota may be mediated via the “oral-gut axis,” wherein oral bacteria migrate through saliva swallowing from the oropharynx or oral digestive sites and ectopically colonize the gut, triggering inflammatory responses (MaChado et al., 2021). This process may disrupt the normal equilibrium of gut microbiota, leading to gut microbial dysbiosis (Bao et al., 2022; Wu et al., 2022). Additionally, these translocated bacteria may modify oral microbial composition during oral transit before entering the gastrointestinal tract. These findings suggest a bidirectional communication between altered microbial communities in the oral and gut ecosystems, influencing both niches (Park et al., 2021).

In this experiment, SourceTracker analysis targeting the genus Prevotella was performed on samples from the HG and COG groups. The results showed that the transmission effect in the COG group was significantly greater than that in the HG group, further confirming the role of the “oral-gut axis” in disease development. Specifically, the migration of Prevotella from the oral cavity to the gut was more pronounced in the COG group, indicating that in the comorbidity of dental caries and obesity, the oral-gut barrier is altered, making it easier for oral bacteria to colonize the gut. Prevotella is a common oral bacterium, Gram-negative and anaerobic, and is widely found in the human oral cavity, gastrointestinal tract, and urogenital tract (Research progress on Prevotella copri in the gut and its association with inflammatory diseases, n.d.). Studies have shown that Prevotella can migrate from the oral cavity to the gut via the oral-gut axis and colonize the gut, potentially triggering inflammatory responses, disrupting gut barrier function, and leading to systemic inflammation and metabolic disorders (Yamazaki and Kamada, 2024). This migration and colonization may affect the host’s health through various mechanisms.

## 5 Conclusion

This study explored the impact of psychological factors on adolescent caries-obesity comorbidity from the “oral-gut-brain axis,” yielding key conclusions:

1. A complex interrelation exists among adolescent caries, obesity, and psychological disorders, forming a health problem network.
2. Oral and gut microbiota play crucial roles in the development of these conditions. 16S rRNA sequencing revealed significant differences in oral and gut microbiota at phylum and genus levels among adolescents in different health states, with microbial diversity and composition closely linked to health.
3. Non-targeted metabolomics showed significant metabolite changes between groups, suggesting oral and gut microbial metabolites may influence brain function via the “gut-brain axis” and “oral-brain axis,” potentially impacting neuropsychiatric diseases.
4. Source tracker analysis confirmed the “oral-gut axis” role in disease, with oral bacteria from caries-obesity patients colonizing the gut and influencing its microbiota, contributing to disease development.

## Data Availability

The data presented in the study are deposited in the NCBI repository, accession number PRJNA1305107\MTBLS12873.
